# Clinical and cardiovascular magnetic resonance factors associated with elevated neutrophil-to-lymphocyte ratio in patients with heart failure: an analysis of a single-centre, prospective registry

**DOI:** 10.1136/bmjopen-2025-101707

**Published:** 2025-08-27

**Authors:** Patrick Thompson, Millie Duckett, Raluca Tomoaia, Wasim Javed, Thomas Anderton, Erica Dall’Armellina, Eylem Levelt, Christopher E D Saunderson, Peter Kellman, John Pierre Greenwood, Sven Plein, Richard Cubbon, Peter P Swoboda

**Affiliations:** 1 Leeds Institute of Cardiovascular and Metabolic Medicine, University of Leeds, Leeds, UK; 2 Leeds Teaching Hospitals NHS Trust, Leeds, UK; 3 Laboratory of Cardiac Energetics, National Heart, Lung, Blood Institute, Bethesda, Maryland, USA; 4 Baker Heart and Diabetes Institute, Melbourne, Victoria, Australia; 5 University of Melbourne and Monash University, Melbourne, Victoria, Australia

**Keywords:** Heart failure, Magnetic resonance imaging, Inflammation

## Abstract

**Abstract:**

**Objectives:**

The neutrophil-to-lymphocyte ratio (NLR) is defined as the ratio of neutrophils to lymphocytes measured in the full blood count. It has been studied across a range of conditions including cancer, sepsis and stroke. It has been proven that in patients with heart failure (HF) with reduced ejection fraction (HF-rEF), an elevated NLR reflects a higher risk of adverse outcomes. The aim of this study is to identify which clinical or cardiovascular magnetic resonance (CMR) factors are associated with an elevated NLR in patients with HF-rEF.

**Design:**

This study was an analysis of the MATCH registry (MyocArdial Tissue Characteristics in patients with heart failure according to glycaemic status), a prospectively recruited registry of patients presenting with a new diagnosis of HF and referred to our centre for a CMR.

**Setting:**

Single-centre study performed in the Advanced Imaging Centre, Leeds General Infirmary, UK. Data collection took place between February 2018 and March 2023.

**Participants:**

Patients (N=605) with newly diagnosed HF-rEF referred for CMR.

**Intervention:**

Participants underwent clinical assessment, medication review, full blood count and CMR on the same day. The CMR protocol included quantitative assessment of myocardial blood flow at stress and rest, late gadolinium enhancement imaging and parametric mapping. Association between NLR, clinical and CMR parameters was examined by linear regression.

**Results:**

The factors which were found to be significantly associated with an elevated NLR were age, atrial fibrillation, N-terminal pro-B-type natriuretic peptide (NT-proBNP), presence of ischaemic fibrosis and myocardial perfusion reserve (MPR). There was no association between NLR and CMR markers of inflammation (native T1 and T2). On multiple regression after correction for age, atrial fibrillation, New York Heart Association classification and left ventricular ejection fraction, the association between NLR and presence of ischaemic fibrosis (coefficient 0.68, 95% CI 0.23 to 1.12, p=0.003) and NT-proBNP (coefficient 0.0002, 95% CI 0.00006 to 0.0003, p=0.002) remained significant. However, the association between MPR was no longer significant (coefficient −0.09, 95% CI −0.28 to 0.09, p=0.330).

**Conclusion:**

In patients with HF with elevated NLR, these findings show an association with worsening congestion (NT-proBNP) and occult coronary artery disease (ischaemic fibrosis). Further studies are required to demonstrate if this accounts for the adverse prognosis. Importantly, there was no association between myocardial inflammation or oedema and NLR.

**STRENGTHS AND LIMITATIONS OF THIS STUDY**

- Large prospectively recruited study specifically designed to reflect a real-life cohort of patients with heart failure.
- A complete physical assessment, blood and cardiovascular magnetic resonance scan was completed in one visit to minimise inconvenience to patients.
- This is an observational study and caution should be applied when forming conclusions from observational data.
- This study was performed in a single centre and thus results may not be applicable across different population groups.

## Introduction

 Heart failure (HF) is estimated to affect almost 1 million people in the UK^1^ and more than 64 million people worldwide.^2^ In developed countries, the overall prevalence of HF is conservatively estimated as 1–3%, with the prevalence increasing with age and thought to affect greater than 10% of those over 70 years of age and 30% of those over 85.^3^

The neutrophil-to-lymphocyte ratio (NLR) is an inexpensive, simple and readily available test that has grown in popularity in recent years as a measure of stress and inflammation. It is defined as the ratio of neutrophils to lymphocytes as measured in the full blood count. The NLR will rise in response to neutrophilia or lymphopenia. Neutrophilia is mainly regulated by the innate immune system and is the immediate response to invading pathogens via chemotaxis, phagocytosis and degranulation. Conversely, lymphocytes are more associated with adaptive immunity, and lymphopenia may be caused by conditions such as nutritional deficiency, immunosuppression or haematological malignancy.

A number of inflammatory conditions have previously been investigated and found to be associated with an elevated NLR, including bacterial infection,^4^ trauma,^5^ acute stroke^6^ or malignancy.^7^ There is no agreed normal range of NLR; however, values above 3.0 or less than 0.7 can be considered pathological in adults.^8^ It has been well established that elevated NLR is associated with higher all-cause mortality in the general population and specifically mortality due to heart disease, respiratory disease and kidney disease.^9 10^ NLR has also been used for the stratification of cancer and correlates with the tumour size, stage and metastatic potential and has an independent prognostic role in survival. Likewise, in sepsis and severe infection, the downward trend of an elevated NLR is associated with improved survival.^8^

With regard to heart disease, it has been demonstrated that in HF, an elevated NLR is significantly associated with worse outcomes including all-cause mortality, HF-related admissions and cardiovascular death.^11^ Further, NLR has been shown to be a better predictor of mortality than absolute neutrophil or lymphocyte count in patients admitted with acute decompensated HF.^12^ Cho *et al*^13^ found that a raised NLR is an independent predictor of mortality following a HF-related hospital admission regardless of the left ventricular ejection fraction (LVEF). For coronary artery disease (CAD), Shah *et al*^14^ found that in the general population, an NLR>4.5 independently predicts CAD-related mortality and that those in the intermediate risk category of the Framingham Risk Score should be reclassified upward. A raised NLR predicts plaque vulnerability and severe stenosis and is associated with larger infarcts and worse long-term outcomes in patients with acute coronary syndrome.^15^

Cardiovascular magnetic resonance (CMR) is considered the gold standard imaging modality for non-invasive assessment of cardiac function, tissue characterisation and viability.^16^ Both the European Society of Cardiology (ESC)^17^ and the American Heart Association^18^ guidelines recommend CMR to assess myocardial structure and function in HF. Patterns of fibrosis and perfusion characteristics by CMR can provide insight into the aetiology of HF and differentiate between ischaemic and non-ischaemic causes, and parametric mapping CMR can detect myocardial inflammation and oedema in myocarditis and inflammatory cardiomyopathies.^19^

The aim of this study was to identify which clinical and CMR factors are associated with an elevated NLR in a HF population with ejection fraction<50%. These patients are potentially at highest risk of adverse outcomes and should be appropriately recognised to ensure optimal clinical care.

## Methods

### Study cohort

We performed an analysis of patients from the MATCH registry (MyocArdial Tissue Characteristics in patients with heart failure according to glycaemic status) who had recently been diagnosed with HF and referred for CMR to investigate the cause. MATCH is a prospectively recruited registry designed to use CMR to investigate patterns of fibrosis and myocardial blood flow in patients with HF both with and without diabetes. Between February 2018 and March 2023 over 600 patients have been added to this registry providing a wealth of data which has been used to inform a number of recent research projects in this field.^20 21^

Participants were deemed ineligible if they had one of the following: LVEF>50% at initial echocardiographic assessment, an established diagnosis of CAD found either invasively or non-invasively (>70% stenosis of a major coronary artery or >50% stenosis of the left main stem on coronary angiography, prior myocardial revascularisation, history of myocardial infarction or presence of typical anginal chest pain), known congenital heart disease or structural heart disease, suspected myocarditis, active infection or significantly impaired renal function not suitable for MRI contrast.

### Clinical assessment

Participants referred from across the region attended our centre for a single appointment which consisted of a comprehensive clinical assessment and a CMR scan. Clinical evaluation comprised gathering demographic data, determination of the New York Heart Association (NYHA) functional class, documenting comorbidities and current medications and assessing for the presence of cardiovascular risk factors. Blood was drawn at the time of intravenous cannulation to measure the full blood count, glycated haemoglobin(HbA1c) and N-terminal pro-B-type natriuretic peptide (NT-proBNP). NLR was determined from the full blood count by dividing the neutrophil count by the lymphocyte count.

### CMR acquisition

**Figure 1:**
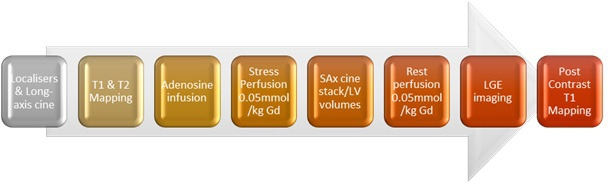
Cardiovascular magnetic resonance acquisition protocol. LGE, late gadolinium enhancement; LV, left ventricular; SAx, short axis.

CMR was performed on a 3T MRI scanner (Siemens Magnetom Prisma, Erlangen, Germany). All patients were advised to abstain from caffeine for at least 24 hours prior to scanning. The CMR protocol involved: cine imaging in long and short axis; adenosine stress perfusion with quantitative assessment of myocardial blood flow (MBF) at rest and stress; motion corrected late gadolinium enhancement (LGE) with phase sensitive inversion recovery sequences in both long and short axis; the addition of dark blood LGE images when required for determination of subendocardial fibrosis; and parametric mapping including native T1, post-contrast T1 and T2 imaging (figure 1).

Pharmacological stress was achieved using an adenosine protocol. This was initially infused at 140 µg/kg/min for at least 3 min. In the event of an inadequate stress response (defined as the absence of physical symptoms or less than a 10 beats per minute increase in heart rate), the adenosine dose was uptitrated to a maximum dose of 210 µg/kg/min. Perfusion images were acquired in three short-axis slices using a T1-weighted saturation recovery gradient echo sequence after administering gadolinium-based contrast.

### Image analysis

Image analysis was performed using cvi42 (Circle Cardiovascular Imaging, Calgary, Canada). Left ventricular (LV) volumes and mass were calculated by manual contouring of endocardial and epicardial borders at end systole and end diastole, excluding trabeculations. All measurements were indexed to body surface area.

LGE was identified if visible on two orthogonal planes. The pattern and location of scar was recorded with ischaemic LGE defined as involving the subendocardium in a typical coronary artery distribution.

T1 mapping values and extra cellular volume (ECV) were calculated using cvi42 (Circle Cardiovascular Imaging, Calgary, Canada). A single mid-ventricular slice with 15% offset to avoid blood pool contamination was taken for both pre and post contrast sequences. These results, alongside the haematocrit (from the full blood count), were used to calculate ECV.

Inducible ischaemia was defined as the presence of a visual perfusion defect involving more than one myocardial segment on stress perfusion images but not present at rest or in a matching segment on LGE imaging. Perfusion mapping was performed using the Gadgetron streaming software image reconstruction framework, giving a numerical value of perfusion in each myocardial segment. Individual segments were compared at rest and stress to investigate for any evidence of regional ischaemia in addition to the recording of global perfusion values at both rest and stress.

### Statistical analysis

Continuous variables were reported as mean±SD or median (IQR). Categorical variables were reported as frequencies and percentages. For comparison, patients were split into one of three tertiles. This was performed using the MedCalc automated function to generate a new categorical variable (Tertile 1–3) categorising patients based on their NLR result.

Continuous variables including patient demographics, clinical and CMR data were compared using analysis of variance when normally distributed and Kruskal-Wallis otherwise. Categorical data was analysed using the χ^2^ test. Univariate and multivariate multiple regression analysis was conducted to assess the relationship between NLR and both clinical and CMR variables in each group. Statistical analysis was conducted using MedCalc Statistical Software V.22.016 (MedCalc Software, Ostend, Belgium; http://www.medccalc.org; 2023). A p value of <0.05 was deemed significant.

### Patient and public involvement

None.

## Results

Overall, 654 patients were recruited from the MATCH registry during the study duration. Of these, 49 were excluded because an NLR result was not recorded.

Of the remaining study population (n=605 patients), 64% were male with a mean age of 67 (58.3–76) years. The mean ejection fraction from baseline referral echo data was 32.1% (±13.0%); 28% of the study population had a HF-related hospital admission within the past year. At the time of CMR assessment, the median NT-proBNP was 561 (166–1655 pg/mL) and the CMR derived mean ejection fraction was 39.7% (±13.1%).

Patient demographics and clinical data of the study population divided by NLR tertile are displayed in table 1. Patients in the highest tertile were significantly older and had higher NT-proBNP. In Tertile 1, the median age was 63 years with an NLR of 1.6 compared with 71 years and NLR 4.38 (p<0.001) in Tertile 3. Between Tertiles 1 and 3, the median NT-proBNP result increased from 422 pg/mL to 863 pg/mL; a significant correlation with p<0.001. Of note, no significant differences were found between groups in terms of gender, comorbidity or HF therapies.

**Table 1:** General characteristics and results of ANOVA/χ^2^ test

	All patients (n=605)	Tertile 1 (n=206)	Tertile 2 (n=198)	Tertile 3 (n=201)	P value
NLR, median (IQR)	2.46 (1.91–3.65)	1.60 (1.25–1.92)	2.47 (2.29–2.79)	4.38 (3.65–5.56)	<0.001
Age (years), median (IQR)	67 (58.3–76)	63 (57–71)	67 (56.3–76)	71 (61–79)	<0.001
Male, n (%)	386 (64)	125 (60.7)	124 (62.6)	137 (68.2)	0.36
Hypercholesterolaemia, n (%)	172 (28.4)	66 (32)	56 (28.3)	50 (24.9)	0.34
Hypertension, n (%)	269 (44.5)	90 (43.7)	85 (42.9)	94 (46.8)	0.6
Diabetes, n (%)	102 (16.9)	30 (14.6)	34 (17.2)	38 (18.9)	0.49
Atrial fibrillation, n (%)	209 (34.6)	67 (32.5)	59 (29.8)	83 (41.3)	0.08
ACEi/ARB, n (%)	471 (77.9)	158 (76.7)	157 (79.3)	156 (77.6)	0.73
Beta-blocker, n (%)	495 (81.8)	165 (80.1)	158 (79.8)	172 (85.6)	0.36
MRA, n (%)	231 (38.2)	74 (35.9)	72 (36.4)	85 (42.3)	0.49
Sacubitril-valsartan, n (%)	58 (9.6)	20 (9.7)	16 (8.1)	22 (10.9)	0.73
SGLT2i, n (%)	62 (10)	19 (9.2)	19 (9.6)	22 (10.9)	0.81
Diuretic, n (%)	266 (44)	76 (36.9)	88 (44.4)	102 (50.7)	0.08
NYHA >2, n (%)	57 (9.5)	19 (9.2)	17 (8.6)	21 (10.4)	0.27
NT-proBNP (pg/mL), median (IQR)	561 (166–1655)	422 (135–1270)	509 (126–1418)	863 (293–2036)	<0.001

ACEi, angiotensin-converting enzyme inhibitors; ANOVA, analysis of variance; ARB, angiotensin receptor blocker; MRA, mineralocorticoid receptor antagonist; NLR, neutrophil-to-lymphocyte ratio; NT-proBNP, N-terminal pro-B-type natriuretic peptide; NYHA, New York Heart Association classification; SGLT2i, sodium-glucose cotransporter-2 inhibitors.

Table 2 demonstrates CMR parameters for each of the groups. Patients in Tertile 3 with higher NLR were significantly more likely to have ischaemic fibrosis by LGE imaging (p=0.0016) and more likely to have significant inducible ischaemia on visual analysis of adenosine stress perfusion imaging (p=0.0112). No significant differences were found in heart size, systolic function, non-ischaemic fibrosis, MBF or T1 or T2 results.

**Table 2:** CMR characteristics and results of ANOVA/χ^2^ test

	All patients (n=605)	Tertile 1 (n=206)	Tertile 2 (n=198)	Tertile 3 (n=201)	P value
LVEDV (mL), mean±SD	214±73	212±74	213±65	217±80	0.82
LVEDVi (mL/BSA), mean±SD	109±36	107±34	109±34	111±40	0.08
LVEF (%), mean±SD	39.7±13.1	40.9±12.6	39.8±12.6	38.52±14.1	0.19
LV mass (g/m^2^), median (IQR)	128 (100–163)	124 (94–164)	125 (102–153)	133 (104–168)	0.26
LVMi (g/BSA), median (IQR)	65 (54–79)	62 (51–79)	64 (54–76)	69 (55–81)	0.08
RVEDV (mL), median (IQR)	148 (122–181)	144 (122–179)	147 (124.3–175.8)	153 (121–184)	0.69
RVEDVi (mL/BSA), median (IQR)	75 (63–90)	74.5 (62–88)	74 (63.5–89)	77 (62–94)	0.55
Non-ischaemic LGE, n (%)	182 (30.2)	57 (27.7)	56 (28.3)	69 (34.3)	0.32
Ischaemic LGE, n (%)	110 (18.3)	29 (14.1)	28 (14.1)	53 (26.4)	0.002
Inducible ischaemia, n (%)	38 (6.3)	13 (6.3)	5 (2.5)	20 (10.0)	0.01
Global stress MBF (mL/min/g), median (IQR)	1.61 (1.28–1.97)	1.62 (1.27–2.00)	1.65 (1.32–1.97)	1.55 (1.24–1.92)	0.33
Global rest MBF (mL/min/g), median (IQR)	0.62 (0.52–0.75)	0.59 (0.51–0.74)	0.63 (0.53–0.72)	0.64 (0.53–0.78)	0.15
MPR, median (IQR)	2.48 (1.93–3.16)	2.55 (1.98–3.19)	2.5 (2.01–3.24)	2.35 (1.79–3.02)	0.19
Native T1 (ms), mean±SD	1325±51	1325±50	1332±50	1330±53	0.27
ECV (%), mean±SD	26.4±4.6	26.4±4.6	26.3±4.7	26.7±4.5	0.70
T2 (ms), mean±SD	42.2±3.9	42±3.4	42±3.3	42.62±4.8	0.22

ANOVA, analysis of variance; BSA, body surface area; CMR, cardiovascular magnetic resonance; ECV, extra cellular volume; LGE, late gadolinium enhancement; LV, left ventricle; LVEDV, left ventricular end-diastolic volume; LVEDVi, indexed left ventricular end‐diastolic volume; LVEF, left ventricular ejection fraction; LVMi, idneaxed left ventricular mass index; MBF, myocardial blood flow; MPR, myocardial perfusion reserve; RVEDV, right ventricular end-diastolic volume; RVEDVi, indexed right ventricular end-diastolic volume.

Univariate linear regression was used to assess the relationship of each factor against NLR. Age, NT-proBNP, ischaemic LGE, atrial fibrillation, LVEF and myocardial perfusion ratio (MPR) were found to have a significant association with the NLR. No significant correlation was found for the other parameters assessed: gender, NYHA, LV size, LV mass, rest and stress MBF, native T1 and T2, non-ischaemic LGE and inducible ischaemia (table 3).

**Table 3:** Univariate logistic regression for the prediction of increased NLR

Independent variables	Coefficient	95% CI	SE	P value	t	Coefficient determination R^2^
Age	0.03	0.02 to 0.05	0.00649	<0.0001	5.124	0.04159
NT-proBNP	0.0002	0.0001 to 0.0003	0.00004	<0.0001	4.539	0.03796
Male sex	0.23	−0.12 to 0.58	0.1774	0.1976	1.290	0.00276
Ischaemic LGE	0.87	0.44 to 1.29	0.2183	0.0001	3.968	0.02558
AF	0.45	0.10 to 0.80	0.1783	0.0112	2.546	0.01067
NYHA	0.25	−0.01 to 0.50	0.1306	0.0586	1.895	0.00600
LVEDVi	0.004	−0.001 to 0.008	0.00237	0.1203	1.556	0.00402
LVEF	−0.013	−0.03 to −0.00005	0.00651	0.0496	−1.968	0.00643
LVMi	0.005	−0.004 to 0.01	0.00428	0.2678	1.109	0.00205
Non-ischaemic LGE	0.03	−0.33 to 0.39	0.1861	0.8655	0.169	0.00005
Inducible ischaemia	0.29	−0.40 to 0.98	0.3512	0.4141	0.817	0.00111
Global stress MBF	−0.13	−0.43 to 0.18	0.1545	0.4122	−0.821	0.00118
Global rest MBF	0.54	−0.19 to 1.27	0.3743	0.1489	1.446	0.00369
MPR	−0.19	−0.37 to −0.002	0.09344	0.0477	−1.985	0.00853
Native T1	0.003	−0.0008 to 0.006	0.00169	0.1305	1.514	0.00388
T2	0.008	−0.04 to 0.05	0.02411	0.7556	0.311	0.00019

AF, atrial fibrillation; LGE, late gadolinium enhancement; LVEDVi, indexed left ventricular end‐diastolic volume; LVEF, left ventricular ejection fraction; LVMi, indexed left ventricular mass; MBF, myocardial blood flow; MPR, myocardial perfusion reserve; NLR, neutrophil-to-lymphocyte ratio; NT-proBNP, N-terminal pro-B-type natriuretic peptide; NYHA, New York Heart Association classification.

Multivariate linear regression was then used to correct for confounding variables. Independent variables were adjusted for age, atrial fibrillation, NYHA and LVEF. These variables were chosen as basic clinical parameters that are routinely recorded. MPR ratio was no longer significant when this adjustment was made, however ischaemic LGE (coefficient 0.68, 95% CI 0.23 to 1.12, p=0.003) and NT-proBNP (coefficient 0.0002, 95% CI 0.00006 to 0.0003, p=0.002) were still found to be statistically significant predictors of an elevated NLR (table 4).

**Table 4:** Multiple regression analysis for the prediction of increased NLR (adjusted for age, AF, NYHA, NT-proBNP)

Independent variables	Coefficient	95% CI	SE	P value	t	r_partial_	r_semipartial_
Ischaemic LGE	0.68	0.23 to 1.12	0.23	0.003	2.990	0.1226	0.1193
MPR	−0.09	−0.28 to 0.09	0.095	0.330	−0.973	−0.0458	0.0449
NT-proBNP	0.0002	0.00006 to 0.0003	0.00005	0.002	3.059	0.1348	0.1313

AF, atrial fibrillation; LGE, late gadolinium enhancement; MPR, myocardial perfusion reserve; NLR, neutrophil-to-lymphocyte ratio; NT-proBNP, N-terminal pro-B-type natriuretic peptide; NYHA, New York Heart Association classification.

## Discussion

In this cohort of patients presenting with HF, both NT-proBNP and the presence of ischaemic fibrosis on CMR were associated with an elevated NLR. Elevated NLR confers a poor prognosis in patients with HF^11^ and our findings suggest that there is an association with silent ischaemic heart disease (as no patients had chest pain, revascularisation or prior myocardial infarction) and/or congestion.

### NLR and myocardial inflammation

The inflammatory hypothesis has been proposed as one of the mechanisms associating NLR and outcomes in HF. It is well known that inflammation is present in patients with HF. It has been shown that patients with HF have elevated levels of pro-inflammatory cytokines and increased levels of circulating proteolytic enzymes such as myeloperoxidase, acid phosphatase and elastase.^11 12^ Regardless of the aetiology of myocardial injury, there is the same inflammatory response: capillary dilatation and hyperaemia of the vascular bed; increased vascular permeability with capillary leak and oedema; subsequent myocyte cell injury and death; expansion of the extracellular space; and ultimately collagen deposition with scar formation.

International recommendations^19^ propose the use of T1 and T2 mapping for the detection of myocardial inflammation. Inflamed myocardium causes prolongation of T1 and T2 relaxation times and mapping can be used for quantification. T2 imaging is very sensitive to myocardial oedema, specifically the extracellular water content and for this reason is of particular value in acute oedema. T1 relaxation, conversely, is less specific for active inflammation and may be prolonged in regions of increased intracellular and extracellular water content or fibrosis.

It is known that T2 relaxation times and NLR are both increased during acute inflammation;^8^ however, in this study, no significant correlation was found between the two. Likewise, no significant correlation was found with T1 or ECV. Of note, increasing age was associated with an elevated NLR and this may be due to immunosenescence-age-related changes in the bone marrow with a reduction in myeloid lymphocyte production resulting in an elevated NLR without significant inflammation.^22^

Interestingly, the fact that there was a significant correlation of NLR with ischaemic fibrosis may suggest there is something specific about the response to previous infarct rather than fibrosis in general that influences NLR. However, overall, no correlation was found between NLR and the results of T1 or T2 parametric mapping and our findings do not support the hypothesis that increased NLR is associated with diffuse myocardial inflammation.

### NLR and congestion

A second hypothesis for elevated NLR in HF is that NLR reflects a systemic stress response in the presence of HF activating the hypothalamic-pituitary-adrenal axis and increasing sympathetic tone. This increases cortisol production, which in turn increases the number and function of granulocytes while reducing the number of lymphocytes due to cell death by oxidative stress.^13 23^

Our finding of a significant association between NLR and NT-proBNP, even after correction for baseline factors, is compatible with this observation. Natriuretic peptides are secreted from the myocardium into circulation in response to increased wall tension.^24^ It is this wall shear stress that exposes the cells to increased biomechanical strain which can ultimately lead to an inflammatory response.^2^

NT-proBNP is recommended by ESC guidelines as a baseline investigation when HF is suspected.^17^ In addition, it can also be used to evaluate prognosis or the response to treatment.^25^ Although NLR correlates well with NT-proBNP, an elevated NLR has been found independently to be a risk factor for poorer outcomes in HF,^23^ and perhaps these two biomarkers could be used synergistically for greater prognostic value.

It has been observed that lymphocytopenia is associated with increased mortality in HF.^13 26^ In addition to neurohumoral activation and cortisol release other potential mechanisms causing lymphocytopenia include the downregulation of lymphocyte proliferation secondary to inflammatory cytokine activation and enteric losses of lymphocytes secondary to elevated ventricular filling pressures.

### NLR and silent myocardial infarction

Atherosclerosis is a chronic inflammatory condition.^12 27^ It was observed in the CANTOS trial^28^ that in patients with a history of myocardial infarction the use of the monoclonal antibody canakinumab to reduce inflammation by targeting interleukin 1-B reduced the incidence of cardiovascular events. Similarly, Svensson *et al*^29^ investigated canakinumab in patients with clonal haematopoiesis of indeterminate potential (CHIP) and found even greater benefits in this population. CHIP is a pro-inflammatory condition defined as a premalignant myeloid disorder characterised by an age-dependent acquisition of leukaemia associated mutations in peripheral blood which is associated with significantly higher levels of atherosclerosis^30^ and elevated NLR.^31^

**Figure 2:**
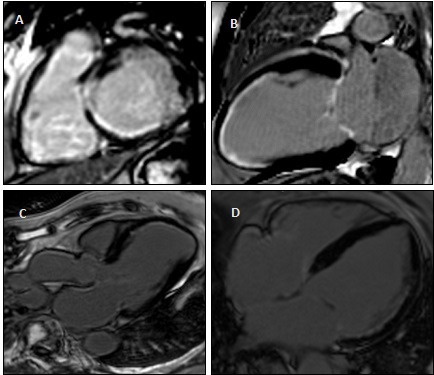
Examples of silent myocardial infarction on cardiovascular magnetic resonance late gadolinium enhancement imaging. (**A**) Basal inferior and inferolateral infarction. (**B**) Mid anterior and apical infarction. (**C**) Mid anteroseptal and apical infarction. (**D**) Basal and mid anterolateral infarction.

Given our observation that NLR is increased in patients presenting with HF and silent myocardial infarction (ischaemic fibrosis detected on CMR in the absence of chest pain, revascularisation or prior history of myocardial infarction—figure 2), clinicians should consider coronary assessment in patients with HF of unknown aetiology and elevated NLR particularly in the presence of cardiac risk factors.

### Study limitations

The design of this single centre study was to use real world data and reflects current clinical practice in our region. To draw conclusions for different populations further larger and randomised studies would be required. There was no standardisation of who was referred for MRI resulting in a possible selection bias. Nor was there standardisation in the timing of referral or prescribing of HF therapies resulting in the fact that HF therapy was likely not optimised in all patients. NLR was only measured once per patient and was taken at the time of attendance for MRI scanning. Patients were not screened for concurrent illness or comorbidities such as a haematological malignancy that may have skewed the NLR result. In addition, other markers of inflammation such as CRP or procalcitonin were not measured. Finally, this was an observational study and caution should always be applied when forming conclusions from observational data which can be prone to confounding or unintentional bias.

## Conclusion

NLR is increasingly recognised as a marker of adverse risk in patients with HF. This prospective study of 605 patients with HF with reduced ejection fraction has identified that NLR is significantly associated with NT-proBNP levels and presence of ischaemic fibrosis. Importantly, there was no association between myocardial inflammation or oedema (measured as T1 and T2) and NLR. Further research is required to investigate if the adverse prognosis associated with elevated NLR in HF is due to worsening congestion or occult CAD rather than diffuse myocardial inflammation.

## Data Availability

Data are available upon reasonable request.

## References

[1] Barasa A, Schaufelberger M, Lappas G (2014). Heart failure in young adults: 20-year trends in hospitalization, aetiology, and case fatality in Sweden. Eur Heart J.

[2] Savarese G, Becher PM, Lund LH (2023). Global burden of heart failure: a comprehensive and updated review of epidemiology. Cardiovasc Res.

[3] Seferović PM, Jankowska E, Coats AJS (2020). The Heart Failure Association Atlas: rationale, objectives, and methods. Eur J Heart Fail.

[4] Huang Z, Fu Z, Huang W (2020). Prognostic value of neutrophil-to-lymphocyte ratio in sepsis: A meta-analysis. Am J Emerg Med.

[5] Park JM (2017). Neutrophil-to-lymphocyte ratio in trauma patients. J Trauma Acute Care Surg.

[6] Li W, Hou M, Ding Z (2021). Prognostic Value of Neutrophil-to-Lymphocyte Ratio in Stroke: A Systematic Review and Meta-Analysis. Front Neurol.

[7] Lee PY, Oen KQX, Lim GRS (2021). Neutrophil-to-Lymphocyte Ratio Predicts Development of Immune-Related Adverse Events and Outcomes from Immune Checkpoint Blockade: A Case-Control Study. Cancers (Basel).

[8] Zahorec R (2021). Neutrophil-to-lymphocyte ratio, past, present and future perspectives. *Bratisl Lek Listy*.

[9] Song M, Graubard BI, Rabkin CS (2021). Neutrophil-to-lymphocyte ratio and mortality in the United States general population. Sci Rep.

[10] Fest J, Ruiter TR, Groot Koerkamp B (2019). The neutrophil-to-lymphocyte ratio is associated with mortality in the general population: The Rotterdam Study. Eur J Epidemiol.

[11] Vakhshoori M, Nemati S, Sabouhi S (2023). Neutrophil to lymphocyte ratio (NLR) prognostic effects on heart failure; a systematic review and meta-analysis. BMC Cardiovasc Disord.

[12] Sorriento D, Iaccarino G (2019). Inflammation and Cardiovascular Diseases: The Most Recent Findings. Int J Mol Sci.

[13] Cho JH, Cho HJ, Lee HY (2020). Neutrophil-Lymphocyte Ratio in Patients with Acute Heart Failure Predicts In-Hospital and Long-Term Mortality. J Clin Med.

[14] Shah N, Parikh V, Patel N (2014). Neutrophil lymphocyte ratio significantly improves the Framingham risk score in prediction of coronary heart disease mortality: insights from the National Health and Nutrition Examination Survey-III. Int J Cardiol.

[15] Agarwal R, Aurora RG, Siswanto BB (2022). The prognostic value of neutrophil-to-lymphocyte ratio across all stages of coronary artery disease. Coron Artery Dis.

[16] Piersson AD (2016). Essentials of cardiac MRI in clinical practice. J Cardiovasc Magn Reson.

[17] McDonagh TA, Metra M, Adamo M (2021). 2021 ESC Guidelines for the diagnosis and treatment of acute and chronic heart failure. Eur Heart J.

[18] Heidenreich PA, Bozkurt B, Aguilar D (2022). 2022 AHA/ACC/HFSA Guideline for the Management of Heart Failure: A Report of the American College of Cardiology/American Heart Association Joint Committee on Clinical Practice Guidelines. Circulation.

[19] Ferreira VM, Schulz-Menger J, Holmvang G (2018). Cardiovascular Magnetic Resonance in Nonischemic Myocardial Inflammation: Expert Recommendations. J Am Coll Cardiol.

[20] Tomoaia R, Harrison P, Bevis L (2024). CMR characterization of patients with heart failure and left bundle branch block. Eur Heart J Imaging Methods Pract.

[21] Goh ZM, Javed W, Shabi M (2023). Early prediction of left ventricular function improvement in patients with new-onset heart failure and presumed non-ischaemic aetiology. Open Heart.

[22] Pang WW, Price EA, Sahoo D (2011). Human bone marrow hematopoietic stem cells are increased in frequency and myeloid-biased with age. Proc Natl Acad Sci U S A.

[23] Curran FM, Bhalraam U, Mohan M (2021). Neutrophil-to-lymphocyte ratio and outcomes in patients with new-onset or worsening heart failure with reduced and preserved ejection fraction. ESC Heart Fail.

[24] Van Linthout S, Tschöpe C (2017). Inflammation - Cause or Consequence of Heart Failure or Both?. Curr Heart Fail Rep.

[25] Hartmann F, Packer M, Coats AJS (2004). Prognostic impact of plasma N-terminal pro-brain natriuretic peptide in severe chronic congestive heart failure: a substudy of the Carvedilol Prospective Randomized Cumulative Survival (COPERNICUS) trial. Circulation.

[26] Petramala L, Milito C, Sarlo F (2024). Clinical impact of transient lymphopenia. Clin Exp Med.

[27] Guo X, Ma L (2023). Coronary artery disease, Vol 34.

[28] Ridker PM, Everett BM, Thuren T (2017). Antiinflammatory Therapy with Canakinumab for Atherosclerotic Disease. N Engl J Med.

[29] Svensson EC, Madar A, Campbell CD (2022). TET2-Driven Clonal Hematopoiesis and Response to Canakinumab: An Exploratory Analysis of the CANTOS Randomized Clinical Trial. JAMA Cardiol.

[30] Jaiswal S, Natarajan P, Silver AJ (2017). Clonal Hematopoiesis and Risk of Atherosclerotic Cardiovascular Disease. N Engl J Med.

[31] Larsen MK, Skov V, Kjaer L (2022). Clonal haematopoiesis of indeterminate potential and impaired kidney function-A Danish general population study with 11 years follow-up. Eur J Haematol.

