# Supplementary material for: Clinical and cardiovascular magnetic resonance factors associated with elevated neutrophil-to-lymphocyte ratio in patients with heart failure: an analysis of a single-centre, prospective registry
Source: BMJ Open. 2025 Aug 27;15(8):e101707. doi: 10.1136/bmjopen-2025-101707 (PMC12410601; doi:10.1136/bmjopen-2025-101707)
Supplement: Supplementary file 1 [file bmjopen-2025-101707.reviewer_comments.pdf]

PEER REVIEW HISTORY

BMJ Open publishes all reviews undertaken for accepted manuscripts. Reviewers are asked to complete a checklist review form and are provided with free text boxes to elaborate on their assessment. These free text comments are reproduced below.

ARTICLE DETAILS

Title (Provisional)

Clinical and cardiovascular magnetic resonance factors associated with elevated neutrophil-to-lymphocyte ratio in patients with heart failure: an analysis of a single-centre, prospective registry

Authors

Thompson, Patrick; Duckett, Millie; Tomoaia, Raluca; Javed, Wasim; Anderton, Thomas; Dall'Armellina, Erica; Levelt, Eylem; Saunderson, Christopher ED; Kellman, Peter; Greenwood, John Pierre; Plein, Sven; Cubbon, Richard; Swoboda, Peter P

VERSION 1 - REVIEW

**Reviewer**	**1**
**Name**	**Rawat, Anurag**
**Affiliation**	**Himalayan Institute of Medical Sciences**
**Date**	**13-Jun-2025**
**COI**	**None**

The manuscript is clearly written and well structured. The abstract is good as it has summarized the study objectives, methods and key findings.A little more detail can be added about NLR values above 3.0 or below 0.7 which are considered as pathological. This is my suggestion to ensure uniformity in writing abbreviations and consider adding some sentences about the clinical utility of NLR in guiding coronary assessment in heart failure patients as this may be helpful for clinician.

This is a single centre study and needs larger randomized study to make it more acceptable for different population groups. There was no fixed protocol for referring or prescribing heart failure therapies and hence heart failure therapies were not optimised. Elevated NLR may be found in other conditions, and they were not properly evaluated. Chances of bias is there as this is single centre observational study.

**Reviewer**	**2**
**Name**	**Jensen, Jesper**

**Affiliation**	**Aarhus University Hospital Department of Cardiology**
**Date**	**13-Jun-2025**
**COI**	**None**

Thomsen and colleagues have presented a paper with an interesting topic. Inflammation in heart failure and atherosclerotic cardiovascular disease is recognised as a risk factor and key driver of these diseases. Neutrophil-to-lymphocyte ratio (NLR) was investigated in a cohort of 605 patients with newly diagnosed heart failure (Ejection fraction<50%) who had CMR conducted. Elevated NLR levels were associated with worsening congestion and ischaemic fibrosis.

As mentioned by the authors, there are several conditions that can cause elevation of the NLR ratio. In addition, this cohort is selected so more information about the indication for having CMR performed would benefit the paper.

1) Can the authors outline the timeline between the blood samples taken and the CMR

2) Was CRP and other blood samples (hemoglobin, thrombocytes, troponins etc.) measured ? These samples must be available from referring departments or hospitals

3) The prognosis or adverse outcome of NLR is mentioned in the introduction and as part of the aim of the study but there is no outcome data presented

VERSION 1 - AUTHOR RESPONSE

Reviewer: 1
Dr. Anurag Rawat, Himalayan Institute of Medical Sciences

1. The manuscript is clearly written and well structured. The abstract is good as it has summarized the study objectives, methods and key findings. A little more detail can be added about NLR values above 3.0 or below 0.7 which are considered as pathological. This is my suggestion to ensure uniformity in writing abbreviations and consider adding some sentences about the clinical utility of NLR in guiding coronary assessment in heart failure patients as this may be helpful for clinician.

Author response: Thank you for the kind comments. The introduction section has been expanded to include more detail about pathological NLR values and guiding coronary assessment.

2. This is a single centre study and needs larger randomized study to make it more acceptable for different population groups. There was no fixed protocol for referring or prescribing heart failure therapies and hence heart failure therapies were not optimised. Elevated NLR may be found in other conditions, and they were not properly evaluated. Chances of bias is there as this is single centre observational study.

Author Response: In the limitations section the following sentence has been added: “To draw conclusions for different populations further larger and randomised studies would be required”. I accept the points that there was no fixed protocol for the prescribing of heart failure therapies, other causes of elevated NLR not fully evaluated and there is a chance of bias with a single centre observational study. This was already acknowledged in the limitations section of the discussion and

has not been amended, however the fact that this was an observational study and a single centre study have been added to the Strengths and Limitations box following the abstract.

Reviewer: 2
Dr. Jesper Jensen, Aarhus University Hospital Department of Cardiology

1. More information about the indication for having CMR performed would benefit the paper.

Author Response: The indication for having a CMR was to investigate the aetiology of heart failure in a cohort of patients with newly diagnosed left ventricular systolic dysfunction and without known coronary artery disease.

The following is included in the methods section: “had recently been diagnosed with HF and referred for CMR to investigate the cause”.

Preserved ejection fraction >50% and coronary artery disease (including how it is defined) have been stated in the exclusion criteria.

2) Can the authors outline the timeline between the blood samples taken and the CMR

Author Response: Patients attended our centre for a single visit, blood sampling was performed at the time of cannulation immediately prior to the CMR.

“Blood was drawn at the time of intravenous cannulation to measure the full blood count, HbA1c and NT-pro BNP.”

3) Was CRP and other blood samples (hemoglobin, thrombocytes, troponins etc.) measured? These samples must be available from referring departments or hospitals.

Author Response: Only a full blood count, HbA1c and NT-pro BNP were collected by our department due to the study protocol and agreed funding. We did not have access to other blood results from all referring hospitals.

3) The prognosis or adverse outcome of NLR is mentioned in the introduction and as part of the aim of the study but there is no outcome data presented

Author response: The aim of the study was to identify risk factors for elevated NLR, it is beyond the scope of this paper to present outcome data. This has been re-worded in the main text to avoid confusion: “The aim of this study was to identify which clinical and CMR factors are associated with an elevated NLR in a heart failure population with ejection fraction <50%”.

VERSION 2 - REVIEW

**Reviewer**	**2**
**Name**	**Jensen, Jesper**
**Affiliation**	**Aarhus University Hospital Department of Cardiology**
**Date**	**19-Jul-2025**

COI

Thank you for the possibility for reviewing the revision of this paper. I find the replies of the authors to both my comments but also the co-reviewer sufficient and adequeate
